# First person – Hanna Berger and Sarah Gerstner

**DOI:** 10.1242/dmm.050813

**Published:** 2024-05-14

## Abstract

First Person is a series of interviews with the first authors of a selection of papers published in Disease Models & Mechanisms, helping researchers promote themselves alongside their papers. Hanna Berger and Sarah Gerstner are co-first authors on ‘ Fbrsl1 is required for heart development in *Xenopus laevis* and *de novo* variants in *FBRSL1* can cause human heart defects’, published in DMM. Hanna is a postdoctoral researcher and Sarah is a PhD student in the lab of Prof. Dr Annette Borchers at Philipps-University Marburg, Germany, investigating early embryonic development with a focus on congenital malformations.


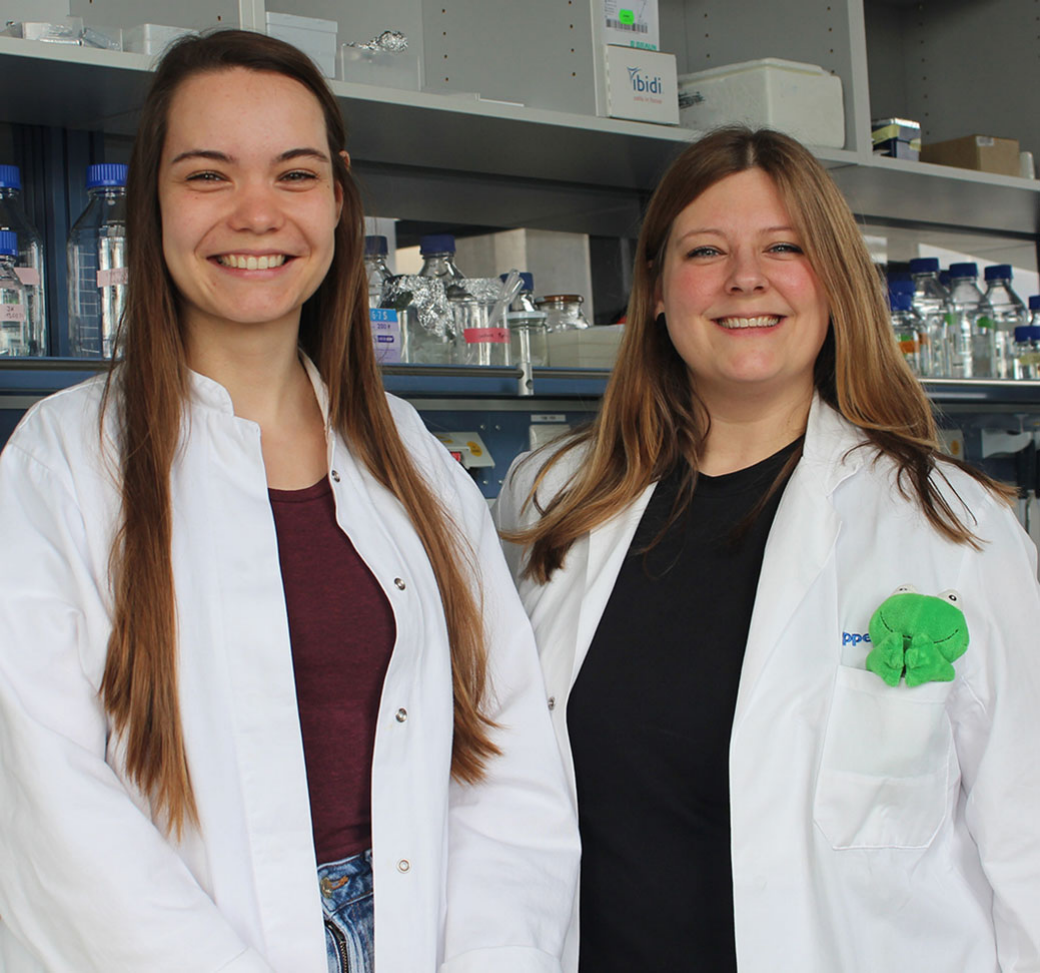


**Hanna Berger (right) and Sarah Gerstner (left)**

**How would you explain the main findings of your paper to non-scientific family and friends?**

**H.B. and S.G.:** Our project started with three unrelated children, who showed overlapping symptoms, but they could not be assigned to any known syndrome. We found a mutation in a specific gene in these patients, the function of which was almost entirely unknown. In our studies, we used the African clawed frog to find out more about the function of this gene. As two of the patients showed heart defects, we focused on the function of this gene in heart development in this part of the project. We found out that the gene is indeed involved in heart development. In particular, it is involved in the development of a specific population of cells that will later give rise to the ventricle and the atria. Additionally, we found evidence, that only a specific part of the gene is important for its function in heart development. This study contributes to a better understanding of patient variants in *FBRSL1* and provides further insights into heart development in general.

I hope that more people with rare diseases will eventually have the chance to learn about their genetic background, how it contributed to their condition and what to expect in the future.

**What are the potential implications of these results for your field of research?**

**H.B.:** It has just recently been described that mutations in Fbrsl1 result in the development of a novel malformation syndrome. Our research aids in the characterization of this syndrome and further helps affected patients and their relatives to gain a better understanding of this condition. Besides, congenital heart defects are amongst the most common congenital malformations, thus studying cardiac development is of major interest. By linking another gene with cardiac development, our study contributes to the broader understanding of developmental processes of the heart.

**S.G.:** Having a case of an unknown congenital condition in my family, I know that patients and their families often do not know what caused the syndrome and how it will affect their lives. Our research will not only uncover the function of a particular gene, but it will also help to develop pipelines to elucidate the function of novel disease-relevant gene variants. So, I hope that more people with rare diseases will eventually have the chance to learn about their genetic background, how it contributed to their condition and what to expect in the future.

**What are the main advantages and drawbacks of the experimental system you have used as it relates to the disease you are investigating?**

**H.B.:** *Xenopus* is a powerful model organism for studying the cardiac system, as many of the genes involved in cardiac development are well characterized and conserved between *Xenopus* and humans. One of the main advantages of *Xenopus* is the abundance of embryos and the availability of techniques for embryonic manipulation, including targeted injection into specific cells that will develop into the particular tissue of interest. However, a disadvantage of *Xenopus* is its allotetraploid genome, which can make genetic manipulation more challenging, for example, using techniques such as CRISPR/Cas9.

**S.G.:** *Xenopus laevis* is a nice model for studying developmental syndromes, because the embryos are easy to assess and manipulate, and are fast in development. Moreover, different steps of heart development are well described in this model organism. Disadvantages include the anatomical differences between frogs and humans, as the frog has a three-chambered heart, whereas the human heart is four-chambered. However, analysing processes like heart development in different systems is important to understand the process as a whole, and as heart development is highly conserved, the frog is a valuable model system to study cardiac development.

**Figure:**
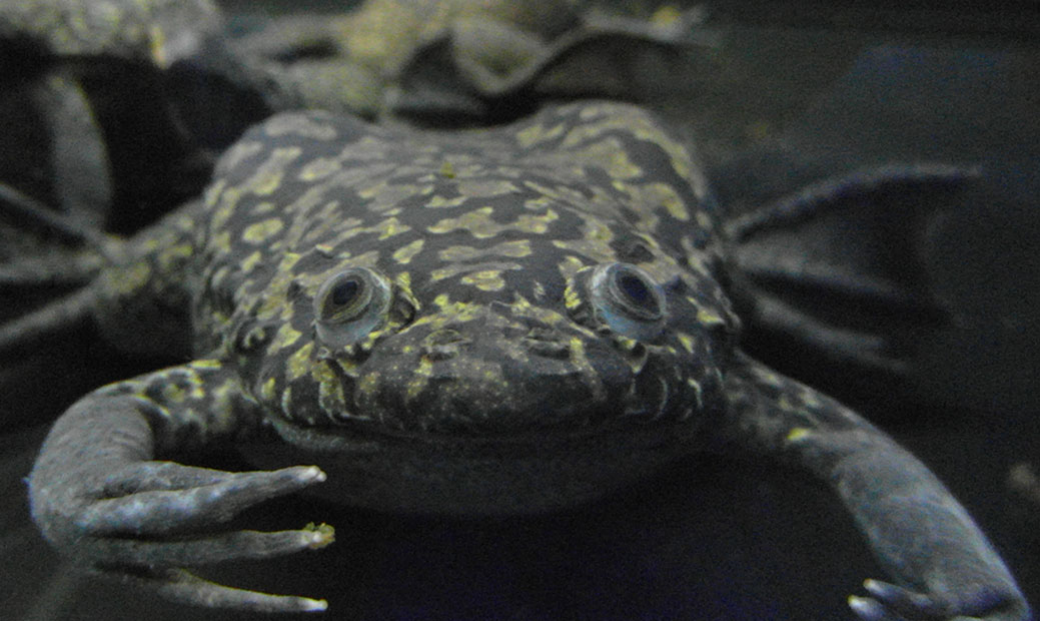
**Adult *Xenopus laevis* frog.** Photo credit: Melanie Bernhardt.

**What has surprised you the most while conducting your research?**

**H.B.:** I was amazed at how long embryos can survive without a functioning heart. This circumstance also enables study of the consequences of longer experimental manipulation compared to other systems.

We think that, in the future, research will develop towards animal-free experiments. There will be more opportunities to grow specific tissues or organs, thereby further reducing the need for research on living animals.

**What do you think is the most significant challenge impacting your research at this time and how will this be addressed over the next 10 years?**

**H.B. and S.G.:** Currently, it is not so easy to visualize the whole heart structure in living embryos, as you have to go deep into the tissue, but still need a high magnification and resolution. Microscopy techniques have improved over the last few years, so we are confident that these tools will soon be accessible to a larger number of laboratories.

Furthermore, we think that, in the future, research will develop towards animal-free experiments. There will be more opportunities to grow specific tissues or organs, thereby further reducing the need for research on living animals.

**What changes do you think could improve the professional lives of scientists?**

**H.B.:** Many scientists are leaving academic careers and switching to the private sector for more security and a better work-life balance. Grant acquisition and fixed-term contracts create uncertainty. It is particularly difficult for families with children who are looking for a permanent position and stability. Increasing the availability of permanent contracts would enhance the attractivity of academic careers.

**S.G.:** I strongly agree with Hanna on this point. Furthermore, I am noticing a trend towards data being compiled in large public databases, which I think is great. This kind of networking will certainly enable faster progress in science.

**What's next for you?**

**H.B. and S.G.:** The Fbrsl1 syndrome has only recently been identified, and there is great potential for further study of this new malformation syndrome. So far, our research indicates a function of Fbrsl1 in heart development as well as in craniofacial and neural development (Ufartes et al., 2020). However, the exact mechanism by which Fbrsl1 affects embryonic development remains unclear.

**H.B.:** I am particularly interested in the development and analysis of birth defects, which has become even more important to me since the birth of my own children. Thus, I am happy to have the privilege to continue working on this exciting project over the next year. However, I am also looking forward to future career opportunities in academia or the private sector.

**S.G.:** As I have some time of my PhD left, I am excited to continue the characterization of the function of this versatile and interesting gene.
